# Serum miR-222-3p as a Double-Edged Sword in Predicting Efficacy and Trastuzumab-Induced Cardiotoxicity for HER2-Positive Breast Cancer Patients Receiving Neoadjuvant Target Therapy

**DOI:** 10.3389/fonc.2020.00631

**Published:** 2020-04-28

**Authors:** Shan Zhang, Yaohui Wang, Yan Wang, Jing Peng, Chenwei Yuan, Liheng Zhou, Shuguang Xu, Yanping Lin, Yueyao Du, Fan Yang, Jie Zhang, Huijuan Dai, Wenjin Yin, Jinsong Lu

**Affiliations:** Department of Breast Surgery, Renji Hospital, School of Medicine, Shanghai Jiaotong University, Shanghai, China

**Keywords:** breast cancer, serum miR-222-3p, neoadjuvant therapy, predictive, prognostic, adverse event

## Abstract

**Background:** We aimed to explore whether the expression of serum miR-222-3p might contribute to early prediction of therapeutic response, clinical outcomes, and adverse events for HER2-positive breast cancer patients receiving neoadjuvant therapy (NAT).

**Methods:** A total of 65 HER2-positive breast cancer patients receiving NAT were analyzed. The concentration of serum miR-222-3p was detected by quantitative real-time PCR. Logistic regression analysis was used to identify the association of serum miR-222-3p with pathological complete response (pCR). The relationship of serum miR-222-3p with disease-free survival (DFS) and overall survival (OS) was examined via log-rank test and Cox proportional hazards analysis. The ordered logistic regression was applied to evaluate the association between serum miR-222-3p and adverse events.

**Results:** The miR-222-3p low group was more likely to achieve pCR [odds ratio (OR) = 0.258, *P* = 0.043]. The interaction between miR-222-3p and presenting Ki67 level was also detected for pCR (OR = 49.230, *P*_*interaction*_ = 0.025). The miR-222-3p low group was correlated with superior DFS (*P* = 0.029) and OS (*P* = 0.0037). The expression of serum miR-222-3p was the independent protective factor for trastuzumab-induced cardiotoxicity (*P* < 0.05) and anemia (*P* = 0.013).

**Conclusions:** Serum miR-222-3p is the potential factor to predict pCR, survival benefit and trastuzumab-induced cardiotoxicity for HER2-positive breast cancer patients receiving NAT.

## Introduction

Neoadjuvant chemotherapy (NAC) is increasingly used in primary breast cancer patients, which not only aims to improve the operability or breast-conservability, but also serves as a good platform for *in-vivo* tests of various drugs (1–3). Trastuzumab, an anti-HER2 monoclonal antibody, is well exemplified in the treatment of HER2-positive breast cancer (4). Manifold data have demonstrated that the addition of trastuzumab to NAC significantly improves the pathological complete response (pCR) rates and thereby results in survival benefit (5–8). However, a majority of HER2-positive breast cancer patients still failed to achieve pCR or even progressed despite trastuzumab-based neoadjuvant therapy (NAT) (9–12). Given the aggressive biological behavior of HER2-positive breast cancer, it hints at a demand to identify potential biomarkers to predict its response to NAT.

On the other hand, adverse events, especially trastuzumab-induced cardiotoxicity, also accompany during or after NAT. The overall incidence of cardiotoxicity was reportedly 3-7% for trastuzumab monotherapy, 13% for trastuzumab with paclitaxel, and as high as 27% for trastuzumab with anthracycline (13, 14). Unfortunately, few reliable biomarkers could help to predict the trastuzumab-induced cardiotoxicity.

Liquid biopsy, as a minimally invasive test, has developed dramatically in recent years. MicroRNAs (miRNAs) belong to a class of noncoding, regulatory, single-stranded RNAs, which have been reported to contribute in early detection of treatment efficacy and adverse reaction (15–23). Our previous research showed that the expression level of miR-222-3p in serum declined after surgery and was an independent prognostic factor for disease-free survival (DFS) in breast cancer (24). Basic studies revealed that miR-222-3p could upregulate HER2 signaling pathway in fulvestrant-resistant breast cancer cells and inhibit the autophagy of cardiac myocytes in mice (25–27). Previous studies have revealed that miR-222-3p was associated with immune invasion and immune resistance in a variety of tumors. Overexpression of miR-222-3p was found to enhance the resistance of tumor cells to tumor infiltrating lymphocytes (TILs) by down-regulating the expression of intercellular cell adhesion molecule-1 (ICAM1) in melanoma, which resulted in ipilimumab (anti-cytotoxic T lymphocyte-associated antigen-4 antibody) resistance in patients with melanoma (28). On the other hand, Ying et al. found that in epithelial ovarian cancer, cancer cell-derived exosomes with high contents of miR-222-3p transferred to the tumor-associated macrophages (TAM) and then induced their polarization to the M2 phenotype via SCOX3/STAT3 pathway (29). The transformation of TAM from M1 to M2 phenotype predicted poor prognosis (30) and contributed to the resistance to anti-HER2/Neu treatment in breast cancer (31). However, it still remains ill-defined whether serum miR-222-3p can serve as a potential biomarker for predicting the response to NAT in HER2-positive breast cancer patients as well as their trastuzumab-induced cardiotoxicity.

On these premises, we hypothesized that the expression of serum miR-222-3p might contribute to early prediction of therapeutic response, clinical outcomes and adverse events for HER2-positive breast cancer patients receiving NAT.

## Materials and Methods

### Study Procedure

All the enrolled HER2-positive breast cancer patients came from two neoadjuvant clinical trials registered as SHPD001 (NCT02199418) and SHPD002 (NCT02221999) in ClinicalTrials.gov. The SHPD001 and SHPD002 trials were verified and authorized by the Independent Ethical Committee of Renji Hospital, Shanghai Jiaotong University. Each patient signed written informed consent.

The eligibility criteria for these two neoadjuvant trials included women aged ≥18 and ≤70 years old with locally advanced invasive breast cancer (T2-4 or N1-3) confirmed independently by two pathologists based on World Health Organization (WHO) classification. All the patients received paclitaxel 80 mg/m^2^ on day 1, 8, 15 and 22 and cisplatin 25 mg/m^2^ on day 1, 8 and 15 every 4 weeks for 4 cycles. For HER2-positive patients, concurrent weekly trastuzumab was given at a loading-dose of 4 mg/kg, followed by maintenance dose of 2 mg/kg, on day 1 for 16 weeks. However, 6 HER2-positive breast cancer patients couldn't afford to receive trastuzumab. In the SHPD002 trial, hormone receptor (HR)-positive patients were randomly assigned to receive preoperative endocrine therapy or not. Endocrine therapy hereinto referred to gonadotropin releasing hormone agonist for premenopausal women and letrozole for postmenopausal counterparts, concurrently with NAC. Tumor assessment was performed every 2 months by physical examination, mammary magnetic resonance imaging (MRI) and ultrasonography. All the patients were required to measure the left ventricular ejection fraction (LVEF) at baseline and every 3 months thereafter. Adverse events were graded according to Common Terminology Criteria for Adverse Events (CTCAE) 4.0. After completion of NAT, the patients underwent surgery. Up until April 2018, 65 HER2-positive breast cancer patients were available for this analysis from these two trials. The results were reported and the analysis was devised according to the Tumor Marker Prognostic Report Recommendation (REMARK) guidelines (32, 33).

### Study Outcome

The primary outcome of SHPD001 and SHPD002 was pCR, which was defined as the absence of tumor in the breast tissues and auxiliary lymph nodes removed at the time of surgery (ypT0 ypN0). The secondary outcomes of SHPD001 and SHPD002 included DFS, overall survival (OS) and adverse events. DFS was defined as the time from surgery until first occurrence of locoregional relapse, contralateral breast cancer, distant metastasis, second primaries and death from any cause. OS was defined as the time from surgery until death from any cause.

### Pathological Examination

Estrogen receptor (ER), progesterone receptor (PR), HER2 and ki67 were evaluated by immunohistochemistry (IHC) in paraffin-embedded tumor samples from core needle biopsy. ER or PR positive was defined as ≥10% of stained cells. HER2-positive was defined as immunohistochemistry (IHC) 3+ or fluorescence *in-situ* hybridization (FISH) amplified according to the American Society of Clinical Oncology (ASCO)/College of American Pathologists (CAP) guideline at that time. Clinical or pathological stage for each patient was determined according to the seventh edition of the American Joint Committee on Cancer staging (AJCC-7).

### Sample Collection

Before NAT, 5 mL peripheral blood was collected in the coagulation-promoting tubes and let stand at 4°C for at least 1 h. Then it was centrifuged at 1,000 × g for 10 min at 4°C to spin down the blood cells. The supernatants were centrifuged at 12,000 × g for 10 min at 4°C to completely remove cellular components. Subsequently, the serum samples were divided into 300 ul/tube in RNA-free EP tubes and stored at −80°C until use (24).

### Extraction of Total RNA

The mirVana PARIS kit (Ambion, Texas, United States) was used to isolate total RNA from 300 μl serum in each patient according to manufacturer's instructions. The extracted RNAs were immediately stored at −80°C until use.

### Quantitative Real-Time PCR

cDNA was obtained by reverse transcription of total RNA using a TaqMan Reverse Transcription Kit (Ambion, Texas, United States). For quantitative real-time PCR, the miRNA-specific TaqMan Small RNA Assays (Ambion, Texas, United States) for miR-222-3p and cel-miR-39 (reference microRNA) were used as described by the manufacturer. The TaqMan primers used for hsa-miR-222-3p (RT002276) and cel-miR-39 (RT000200) were obtained from Applied Biosystems. Briefly, 100 ng of total RNA was reverse transcribed using primers specific to each miRNA target followed by real-time PCR on LightCycler® 480 II (Roche, Mannheim, Germany) using TaKaRa probe qPCR kit (RR390A, TaKaRa, Dalian, China) according to the manufacturer's instructions. The expression of miR-222-3p relative to cel-miR-39 was determined using 2 ^−ΔCT^ method. ΔCt = mean value Ct (miR-222-3p)-mean value Ct (reference miR-39).

### Statistical Analysis

For pCR and survival analysis, the patients were dichotomized into miR-222-3p high (2^−ΔCt^ > 0.03) or low (2^−ΔCt^ ≤ 0.03). Chi-square test or Fisher's test was performed to evaluate the correlation of serum miR-222-3p with clinicopathological characteristics. Logistic regression analysis was used to identify the association of serum miR-222-3p with pCR. Interaction of serum miR-222-3p was also tested with presenting ER or PR status, presenting Ki67 level and the use of neoadjuvant trastuzumab for pCR. The relationship of serum miR-222-3p with DFS and OS was examined via log-rank test and Cox proportional hazards analysis.

For safety analysis, the patients were trichotomized into miR-222-3p low [2^−ΔCt^ ≤ 0.006 (at the 25th percentile)], intermediate (0.006 <2^−ΔCt^ ≤ 0.053) or high [2^−ΔCt^> 0.053 (at the 75th percentile)]. The ordered logistic regression was applied to evaluate the association between serum miR-222-3p and adverse events. The relative and absolute drop of LVEF from baseline were respectively calculated when the LVEF measured at two time points or more were available. The relative drop of LVEF from baseline (rLVEF) was defined as (LVEF_min_-LVEF_baseline_)/LVEF_baseline_, and the absolute drop of LVEF from baseline (aLVEF) as LVEF_min_-LVEF_baseline_. Since grade 3/4 LVEF decrease occurred in only a minority of patients, either rLVEF or aLVEF was divided by its quartile.

The statistical analysis was performed by STATA Statistics SE 14 (Stata Corp LP, College Station, TX, USA). All tests were two-tailed and *P* <0.05 was considered statistically significant.

## Results

### The Association Between the Expression of Serum miR-222-3p and the Clinicopathological Characteristics

The baseline characteristics of all the patients are listed in Table 1. The expression of serum miR-222-3p was associated with presenting clinical N stage (*P* = 0.016) and death (*P* = 0.046). The miR-222-3p low group tended to experience less DFS events (*P* = 0.066). Significant correlation failed to be discovered between serum miR-222-3p and other clinicopathological characteristics.

**Table 1:** The association of serum miR-222-3p with clinicopathological characteristics.

**Characteristics**	***N*** **(%)**		***P* value**
	**miR-222-3p low**	**miR-222-3p high**	
**Age (years)**			
≤ 50	10 (41.67)	16 (39.02)	0.834
>50	14 (58.33)	25 (60.98)	
**Menopausal status**			
Premenopausal	10 (41.67)	15 (36.59)	0.684
Postmenopausal	14 (58.33)	26 (63.41)	
**Presenting clinical T stage**			
T1	2 (4.88)	0 (0.00)	0.402n^*^
T2	7 (17.07)	7 (29.17)	
T3	20 (48.78)	8 (33.33)	
T4	12 (29.27)	9 (37.50)	
**Presenting clinical N stage**			
N0	9 (21.95)	0 (0.00)	0.016^*^
N1	26 (63.41)	23 (95.83)	
N2	2 (4.88)	0 (0.00)	
N3	4 (9.76)	1 (4.17)	
**Presenting ER status**			
Negative	23 (56.10)	14 (58.33)	0.861
Positive	18 (43.90)	10 (41.67)	
**Presenting PR status**			
Negative	18 (43.90)	9 (37.50)	0.613
Positive	23 (56.10)	15 (62.50)	
**Presenting Ki67 level**			
≤ 40%	22 (53.66)	12 (50.00)	0.776
>40%	19 (46.34)	12 (50.00)	
**pCR**			
Yes	22 (53.66)	9 (37.50)	0.208
No	19 (46.34)	15 (62.50)	
**DFS event**			
Yes	3 (7.32)	6 (25.00)	0.066^*^
No	38 (92.68)	18 (75.00)	
**Death**			
Yes	0 (0.00)	3 (12.50)	0.046^*^
No	41 (100.00)	21 (87.50)	

*ER, estrogen receptor; PR, progesterone receptor; pCR, pathological complete response; DFS, disease-free survival*.

* *Fisher's test*.

### The Association Between the Expression of Serum miR-222-3p and the pCR Rate

**Figure 1:**
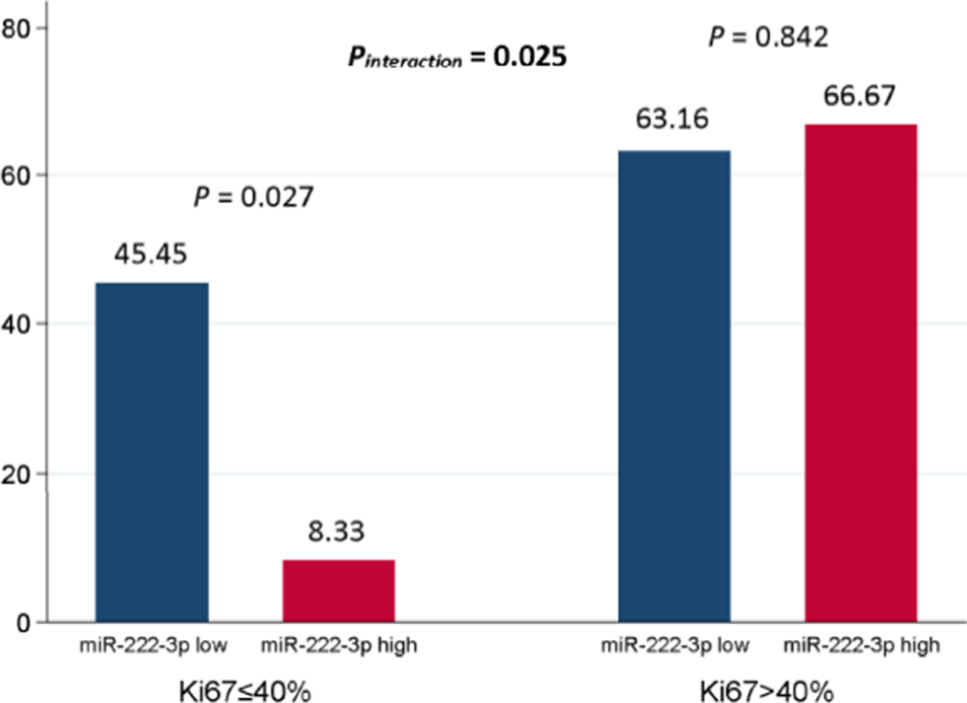
Interaction between serum miR-222-3p and presenting Ki67 level for pCR. The *P* value for interaction was adjusted by serum miR-222-3p level, presenting clinical T stage, presenting clinical N stage, presenting ER status, presenting PR status and presenting Ki67 level in the multivariate logistic analysis. pCR, pathological complete response; ER, estrogen receptor; PR, progesterone receptor.

In general, 31 out of 65 (47.69%) patients reached pCR. In the multivariate analysis, the miR-222-3p low group was more likely to achieve pCR [OR = 0.258, 95% confidence interval (CI): 0.070-0.958, *P* = 0.043]. Meanwhile, patients with lower ER expression (OR = 0.050, 95% CI: 0.007–0.347, *P* = 0.002) and higher Ki67 (OR = 7.155, 95% CI: 1.857–27.566, *P* = 0.004) were much easier to achieve pCR (Table 2). The interaction between miR-222-3p and presenting Ki67 level was also detected for pCR (OR = 49.230, 95% CI: 1.624–1492.078, *P*_*interaction*_ = 0.025; Figure 1). However, no interaction of miR-222-3p was found with presenting ER or PR status and the use of neoadjuvant trastuzumab.

**Table 2:** Multivariate logistic analysis for predictive factors of pCR.

**Characteristics**	**Multivariate logistic analysis**			
	**OR**	**95% CI**		***P* value**
MiR-222-3p (high vs. low)	0.258	0.070	0.958	0.043
T stage (T3-4 vs. T1-2)	0.627	0.142	2.762	0.537
N stage (N2-3 vs. N0-1)	0.174	0.024	1.284	0.086
ER (positive vs. negative)	0.050	0.007	0.347	0.002
PR (positive vs. negative)	4.239	0.743	24.186	0.104
Ki67 (>40% vs. ≤ 40%)	7.155	1.857	27.566	0.004

*pCR, pathological complete response; ER, estrogen receptor; PR, progesterone receptor; OR, odds ratio; CI, confidence interval*.

### Survival Analysis

**Figure 2:**
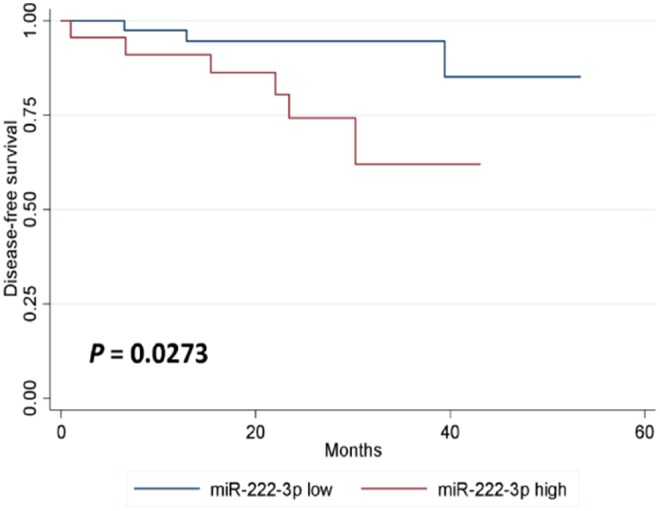
Kaplan-Meier estimates of disease-free survival according to the expression of serum miR-222-3p.

**Figure 3:**
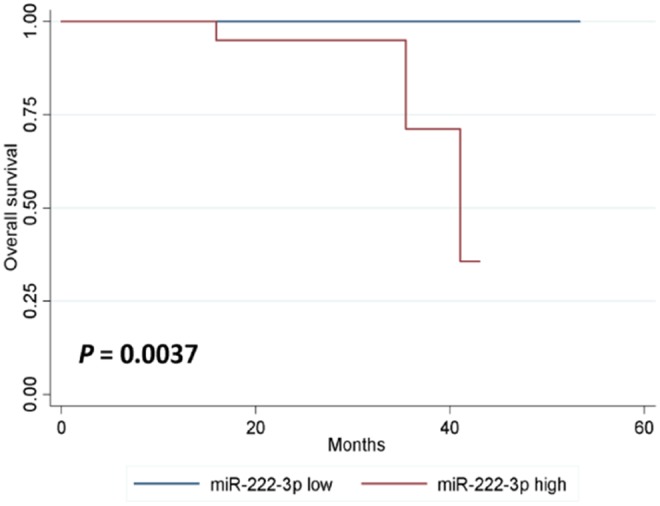
Kaplan-Meier estimates of overall survival according to the expression of serum miR-222-3p.

In either univariate (*P* = 0.0273; Figure 2) or multivariate [hazard ratio (HR) = 5.778, 95% CI: 1.196–27.906, *P* = 0.029; Table 3] survival analysis, superior DFS was seen in the miR-222-3p low group. Furthermore, the lower expression of serum miR-222-3p was also related with better OS (*P* = 0.0037; Figure 3).

**Table 3:** Multivariate survival analysis of serum miR-222-3p and DFS.

**Characteristics**	**Multivariate survival analysis**			
	**HR**	**95% CI**		***P* value**
MiR-222-3p (high vs. low)	5.778	1.196	27.906	0.029
T stage (T3-4 vs. T1-2)	1.172	0.274	5.019	0.831
N stage (N2-3 vs. N0-1)	2.580	0.259	25.736	0.419
ER (positive vs negative)	2.318	0.336	15.969	0.393
PR (positive vs negative)	0.730	0.097	5.474	0.759
Ki67 (>40% vs. ≤ 40%)	0.791	0.202	3.098	0.736

*DFS, disease-free survival; ER, estrogen receptor; PR, progesterone receptor; HR, hazard ratio; CI, confidence interval*.

### The Association Between the Expression of Serum miR-222-3p and Adverse Events

The ordered logistic regression analysis showed that the expression of serum miR-222-3p was the independent protective factor for rLVEF (OR = 0.410, 95% CI: 0.175–0.962, *P* = 0.040; Table 4), aLVEF (OR = 0.394, 95% CI: 0.166–0.937, *P* = 0.035; Table 4), and anemia (OR = 0.408, 95% CI: 0.201–0.828, *P* = 0.013; Table 4).Alopecia (OR = 1.935, 95% CI: 0.893–4.193, *P* = 0.094) and constipation (OR = 2.234, 95% CI: 0.940–5.308, *P* = 0.069) were marginally correlated with the expression of serum miR-222-3p (Table 4).

**Table 4:** The association of serum miR-222-3p and adverse events.

**Adverse event**	**Serum miR-222-3p**			
	**N**	**OR**	**95% CI**	**P**
rLVEF	36	0.410	0.175–0.962	0.040
aLVEF	36	0.394	0.166–0.937	0.035
ALT increase	59	1.340	0.565–3.465	0.467
AST increase	59	0.938	0.452–1.948	0.865
ALP increase	59	1.072	0.148–7.750	0.945
Blood bilirubin increase	59	1.177	0.591–2.342	0.643
Creatinine increase	59	0.820	0.383–1.757	0.610
Leukemia	59	0.852	0.439-1.653	0.635
Neutropenia	59	0.675	0.350–1.230	0.240
Thrombocytopenia	59	1.072	0.148–7.750	0.945
Anemia	59	0.408	0.201–0.828	0.013
Peripheral neuropathy	59	0.938	0.477–1.843	0.852
Nausea	59	1.488	0.728–3.040	0.276
Vomiting	59	1.161	0.551–2.448	0.694
Alopecia	59	1.935	0.893–4.193	0.094
Constipation	59	2.234	0.940–5.308	0.069
Diarrhea	59	1.024	0.523–2.005	0.944
Fatigue	59	0.761	0.368–1.572	0.460

*OR, odds ratio, CI, confidence interval; rLVEF, relative drop of left vetrucular ejection fraction from baseline; aLVEF, absolute drop of left vetrucular ejection fraction from baseline; ALT, alanine aminotransferase; AST, aspartate aminotransferase; ALP, alkaline phosphatase*.

## Discussion

This study, to the best of our knowledge, is the first to report the association between the expression of serum miR-222-3p and the response to NAT in the HER2-positive breast cancer patients. This is also the first time to report the relationship between the serum miR-222-3p expression level and adverse events.

Serum miRNAs were identified as biomarkers to diagnose or predict prognosis in various carcinomas (34, 35). Some basic studies demonstrated that tumor can release miRNAs into circulation and serum miRNAs exist in a remarkably stable form protected from endogenous RNase activity (36, 37), while others indicated the presence of miRNA-enriched exosomes secreted by non-cancer cells such as adipose tissue macrophages and mesenchymal stromal cells (38–40). Therefore, certain miRNAs, with cancer-, tissue- or organ-specific functions, might produce endocrine, paracrine or autocrine effect (41–44) on breast cancer patients, directly or indirectly regulating their response to NAT.

Our data reveal that higher level of serum miR-222-3p was associated with inferior pCR rate, which might be attributed to trastuzumab-resistance. So far, two important pathways might contribute to trastuzumab resistance. Firstly, the activation of phosphatase and tension homologs (PTEN) predicts inhibitory effect of trastuzumab, whereas PI3K pathway activation through PTEN loss and PIK3CA mutation confers trastuzumab resistance in breast cancer (45–49). Secondly, PTEN loss leads to SRC hyperactivation, and SRC inhibition appears to overcome trastuzumab resistance (50–53). On the other hand, miR-222 was reported to promote adriamycin or tamoxifen resistance through PTEN/Akt pathway in breast cancer (54, 55). Consequently, miR-222-3p may also induce trastuzumab resistance by modulating the PTEN/PI3K/Akt pathway and the expression of SRC, which prompts further investigation.

We observed the poor prognosis in the patients with higher level of serum miR-222-3p. An increasing number of researches have demonstrated that miR-222 promotes epithelial-to-mesenchymal transition (EMT) (26, 56, 57), G1/S transition of cell cycle (58) and cell proliferation (59, 60) in breast cancer. Therefore, the expression level of miR-222-3p is responsible for the invasion and metastasis of breast cancer. The prognostic value of miR-222 has also been clarified not only in breast cancer (24, 61) but also in many other malignancies including pancreatic cancer, bladder cancer and glioblastoma (62–66). Furthermore, the anti-miR-222 might improve the chemosensitivity and survival, which provides a novel strategy for breast cancer management (67).

Trastuzumab administration is associated with an increased risk of cardiovascular adverse events (68–70). However, the underlying mechanism remains suspended. Trastuzumab-induced cardiotoxicity, regarded as a type II adverse drug reaction, commonly cause reversible dysfunction without cell loss (71). It generally presents as asymptomatic LVEF decline (72–74). Moreover, LVEF decline frequently improves with drug interruption and resuming trastuzumab after recovery is often feasible (71, 74). This study showed that the impairment of LVEF was associated with the expression level of serum miR-222-3p in this study. MiR-222 has been found to participate in many physiological and pathological processes in the cardiovascular system (75). Additionally, miR-222 is necessary for cardiomyocyte growth induced by exercise and is sufficient to protect against adverse cardiac remodeling after ischemic injury (76, 77). Serum miR-222-3p may play an endocrine role similar to that of hormones, reaching every organ, tissue or cell in the body with blood circulation. When miR-222-3p acts on cancer cells, it plays a role in carcinogenesis and drug resistance, and when it acts on cardiac myocytes, it plays a role in protecting the heart. Therefore, the overexpression of miR-222-3p might prevent the heart from trastuzumab-induced injury.

As far as we know, this is the first time that patients with lower expression level of serum miR-222-3p have been reported prone to anemia after receiving NAT. However, the molecular mechanism has not been clarified. The progressively down-regulating of miR-222-3p was observed during the normal erythropoiesis (78). In addition, miR-222-3p inhibited the expansion of erythroblasts and hematopoietic differentiation via down-regulated c-kit expression and miR-222-3p decreased the expression of BLVRA and CRKL to suppress erythroid differentiation (79–81). Consistent with previous basic studies, our data also showed that the patients with high miR-222-3p level have lower baseline hemoglobin level (OR = 0.07, 95% CI 0.005–0.991, *P* = 0.049). These results suggested that the erythroblasts may be more resistant to chemotherapy in the high miR-222-3p group due to the lower proliferative activity and conversely patients with low level of miR-222-3p might be prone to anemia.

The limitations of this study were as follows. Firstly, the sample size was relatively small. Large-scale studies are needed to confirm the predictive and prognostic value of miR-222-3p. Secondly, some follow-up data are missing on adverse events. Thirdly, as the serum samples were collected before NAT in this retrospective study of prospective trials, it was not possible for us to evaluate the correlation of miR-222-3p changes at different time points during or after NAT with various outcomes including response, survival and safety, which warrants further research.

In conclusion, our study revealed that serum miR-222-3p is the potential factor to predict pCR, survival benefit and trastuzumab-induced cardiotoxicity for HER2-positive breast cancer patients receiving NAT. However, the logic behind it is still open to investigation.

## Data Availability Statement

The datasets generated for this study are available on request to the corresponding author.

## Ethics Statement

The studies involving human participants were reviewed and approved by the Independent Ethical Committee of Renji Hospital, Shanghai Jiaotong University. The patients/participants provided their written informed consent to participate in this study.

## Author Contributions

SZ, YaoW, JL, and WY contributed conception and design of the study. SZ organized the database. YanW, JP, CY, LZ, SX, YL, YD, FY, JZ, and HD contributed acquisition of the data. SZ, YaoW, and WY performed the statistical analysis. SZ and YaoW wrote the first draft of the manuscript. WY and JL revised the manuscript. All authors read and approved the submitted version.

## Conflict of Interest

The authors declare that the research was conducted in the absence of any commercial or financial relationships that could be construed as a potential conflict of interest.
